# Early Neurological Assessment and Long-Term Neuromotor Outcomes in Late Preterm Infants: A Critical Review

**DOI:** 10.3390/medicina56090475

**Published:** 2020-09-15

**Authors:** Domenico M. Romeo, Martina Ricci, Maria Picilli, Benedetta Foti, Giorgia Cordaro, Eugenio Mercuri

**Affiliations:** 1 Pediatric Neurology Unit, Fondazione Policlinico Universitario A. Gemelli, IRCCS, 00146 Rome, Italy; riccimartina.rm@gmail.com (M.R.); mariapicilli@gmail.com (M.P.); benedetta.foti@yahoo.com (B.F.); cordarogiorgia@gmail.com (G.C.); eumercuri@gmail.com (E.M.); 2 Pediatric Neurology Unit, Università Cattolica del Sacro Cuore, 00146 Rome, Italy

**Keywords:** late preterm, neurological outcome, neurological examination, cerebral palsy

## Abstract

*Background and Objectives*: Late preterm (LP) infants (born between 34 and 36 weeks of gestational age) are considered at higher risk of neonatal morbidities, mortality, and neurological impairments than full-term born infants (FT). The aim of this study was to provide a critical review of the literature outlining the different aspects of neurological function reported both in the neonatal period and in the follow up of late preterm infants. *Materials and Methods*: A comprehensive search of the MEDLINE, Embase, PsycINFO, and CINAHL electronic databases was made, using the following search terms: ‘Late preterm infants’, ‘Near term infants’, ‘neurological assessment’, ‘neurological outcome’, ‘neuromotor outcome’, cerebral palsy’, ‘CP’, ‘motor impairment’, including all the studies reporting clinical neurological assessment of LP (including both neonatal period and subsequent ages). *Results*: A total of 35 articles, comprising 301,495 children, were included as fulfilling the inclusion criteria: ten reported neonatal neurological findings, seven reported data about the first two years after birth, eighteen reported data about incidence of CP and motor disorder during the infancy. Results showed a more immature neurological profile, explored with structured neurological assessments, in LP infants compared with FT infants. The LP population also had a higher risk of developing cerebral palsy, motor delay, and coordination disorder. *Conclusion*: LP had a higher risk of neurological impairments than FT infants, due to a brain immaturity and an increased vulnerability to injury, as the last weeks of gestational age are crucial for the development of the brain.

## 1. Introduction

Late preterm (LP) infants are defined as infants born between 34 and 36 weeks of gestational age, and represent the predominant part of infants born prematurely (~70%) [1,2,3].

In literature, they are described as at a relatively low risk of neuromotor impairments compared with other preterm infants born at a lower gestational age and therefore they are often not routinely followed. However, recent studies evidenced that LP are at a higher risk than term-born infants to develop complications such as respiratory distress syndrome, hypoglycemia, hypothermia, prolonged jaundice, and feeding problems [4]. Several studies also reported on neuro-cognitive, social, and behavioral outcomes showing that LP infants have a higher rate of language disorder, Attention Deficit and Hyperactivity Disorder (ADHD), Autism Spectrum Disorder (ASD), school-related problems, Specific Learning Disorder [2], developmental delay or mental retardation [4,5,6,7,8], psychiatric disorders like schizophrenia [8], and worse social competence compared to full-term infants [9]. This is true even in the absence of brain lesions, showing that prematurity per se is a risk factor for neurodevelopmental disease [7].

Only a few studies have systematically analyzed the neuromotor outcome in LP infants, and many of these aspects have often been considered in isolation or in specific age groups. The aim of the present study is therefore to report a critical review of the existing literature on early neurological assessment and neuromotor outcome in both LP newborns and children.

## 2. Materials and Methods

### 2.1. Search Strategy

A comprehensive search was made of the MEDLINE, Embase, PsycINFO, and CINAHL electronic databases. The primary search terms ‘Late preterm infants’ or ‘Near term infants’ were combined with the keywords ‘neurological assessment’ or ‘neurological outcome’ or ‘neuromotor outcome’, ‘cerebral palsy’ or ‘CP’ or ‘motor impairment’.

### 2.2. Inclusion Criteria

Studies were eligible for inclusion if they were written in English. As the aim of the study was to assess the neuromotor outcome in LP infants and children, we included all the studies reporting the following topics: clinical neurological assessment of LP (including both neonatal period and subsequent ages).

### 2.3. Exclusion Criteria

Studies were excluded if they were case reports, or if they assessed progressive and/or neurodegenerative disease, major congenital malformations or genetic disorders, or if they had unusual population demographics including a significant sex imbalance (males < 30%). Studies including cognitive assessments only were also excluded.

### 2.4. Data Extraction and Analysis

The title and abstracts of the studies were independently examined for suitability by two authors (DMR, MR) and critically checked by a third independent reviewer (EM); conflicting viewpoints were discussed until consensus was reached. The selected papers were further subdivided into

(1) studies reporting neonatal neurological examination;
(2) studies reporting data about the neurological examination in the first two years of life;
(3) studies reporting incidence of CP and motor disorders in LP patients.

Demographic variables collected included sex and age when the neurological assessment was performed.

## 3. Results

**Figure 1:**
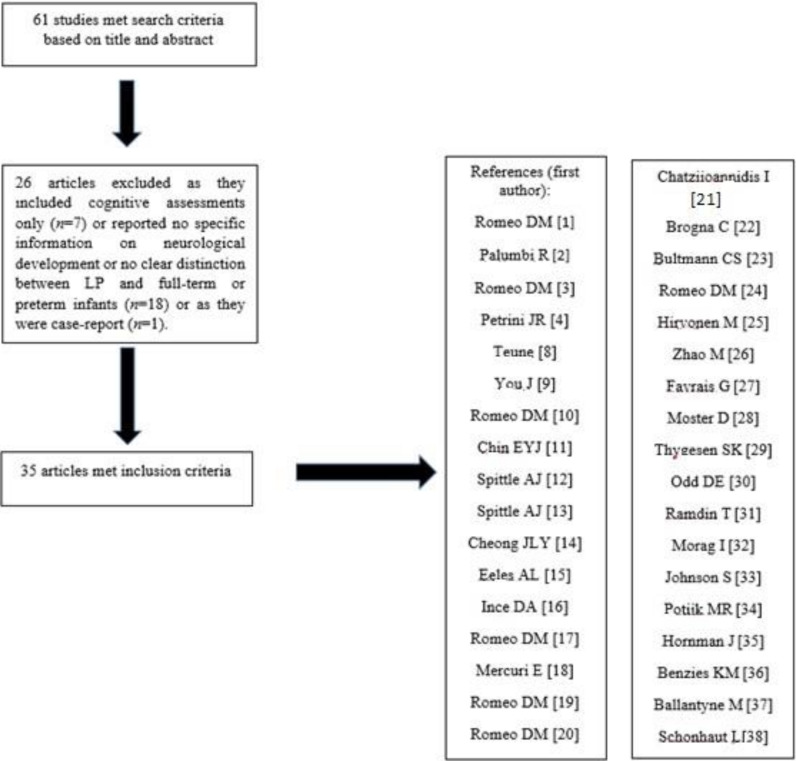
Flowchart of included studies.

A total of 61 studies were initially identified; of them, 26 were excluded as they included cognitive assessments only (*n* = 7) or reported no specific information on neurological development or no clear distinction between LP and full-term or preterm infants (*n* = 18) or as they were case-reports (*n* = 1). The remaining 35 articles, comprising a total of 301,495 children, met the inclusion criteria after a review of the full text including the neurological assessments in LP infants (Figure 1).

### 3.1. Studies Reporting Neonatal Neurological Assessment

Ten studies [3,10,11,12,13,14,15,16,17,18] reported neonatal neurological findings; two were review studies [14,18]. Papers are summarized in Table 1. Eight studies reported structured neurological assessments, the other two studies used different tools to explore specific aspects of neurological function.

All eight of the studies [3,10,11,12,13,14,15] reporting structured neurological assessments used the Hammersmith Neonatal Neurological Examination (HNNE). Two were performed with the aim of establishing the range and frequency distribution of neonatal neurological scores using HNNE in a population of low-risk LP assessed at term-age between 39–41 weeks [10] and during the first three days from birth [3]. In the first study [10], the authors analyzed the results from 375 LP (196 males, 179 females). When examined at term age, infants born at 34 weeks had a more immature profile, in particular in the tone items, as limb flexor tone and head control, Moro reflex and visual orientation, than those born at the age of 35–36 who have a neurological profile similar to full-term (FT) born infants. In the second study [3], the same authors analyzed the results from 118 LP (64 males, 54 females) assessed at three days after birth, reporting a similar distribution of neurological scores among the three GAs for most of the neurological items, with different median scores among LP infants born at 36 weeks and those born at 34 and 35 in only two items (leg recoil and cry).

In both studies, the results showed a wide range of findings for each item that was wider than that reported in full term infants.

Similar results were also obtained by Chin et al. [11] who prospectively studied 212 newborns (79 LP, 43 males, and 36 females, and 133 FT, 57 males, and 76 females) at 12–72 h of life and at term-age (39–41 weeks) using the HNNE, showing similar discrepancies in ‘tone’ and ‘movement’ item between LP and FT both during the first three days and at term-age.

Four of these studies [12,13,14,15] also explored the possibility to combine the neurological assessment with other assessments, such as general movements or structured behavioral assessment.

Spittle et al. [12] reported on a prospective cohort study using the HNNE and general movement assessment (GMA) and the Neonatal Intensive Care Unit Network Neurobehavioral Scale (NNNS). They assessed a population of 129 LP (34 to 36 weeks GA) (64 males and 64 females) 80 moderate preterm (MP) (38 males and 42 females), (born 32 to 33 weeks GA), and 185 FT (born 37 to 42 weeks’ gestation) (97 males and 88 females). Using the HNNE, LP and MP reported similar scores but had lower raw scores with more variability than FT with the exception of flexor posture that showed similar finding into the three group. Using the GMA, LP and MP had a lower rate of normal GM than FT (25–30% vs. 90%). Similar findings were found using the neurobehavioral scale (NNNS), as LP and MP showed lower scores than FT, especially in attention, quality of movement, tone, excitability, reflexes, and stress.

In another study, the same authors examined the association between early HNNE and NNNS and neurodevelopmental outcomes at two years, using the Bayley Scales of Infant and Toddler Development in a cohort of 197 LP and MP (96 males and 101 females) [13]. Better scores on the HNNE, for spontaneous movements, abnormal signs, behavior, and total score and on the NNNS, for attention and regulation scales were associated with better cognitive scores at two years.

The relevance of these newborn neurobehavioral assessments in predicting neurobehavioral outcome at two years was also confirmed in a subsequent review by Cheong et al. [14], reporting suboptimal scores in late preterm infants compared with tern born ones.

Another study using a structured neurological assessment [15] explored the relationships between neurological and neurobehavioral tools at term-equivalent age and brain lesions in a cohort of 196 MP and LP infants (93 males and 103 females) compared to very preterm (VP) newborns. Results showed the presence of a significant relationship between brain MRI abnormalities and suboptimal scores on the NNNS and the HNNE in all groups subdivided according to gestational age at birth, with a stronger relation between brain MRI abnormalities and abnormal GMA in the very preterm group.

The remaining two studies [16,17] explored a specific aspect of neurological performance. Ince et al. [16] evaluated Moro reflex in 35 low-risk LP (13 males and 22 females) and 35 FT (20 males and 15 females), assessed at 48 h of birth. Moro reflex was objectively measured using a the three-axis accelerometer placed on the baby’s wrists and a web camera. They reported a significant difference for latency interval in the complete Moro response with LP infants having shorter duration of Moro response as they were more hyperexcitable, had less flexor tone in the limbs, and less extensor tone in the neck.

In a study performed by Romeo et al. [17], a specific battery to assess neurovisual functions was used in 80 LP (38 males and 42 females) assessed at 48–72 h after birth, and at term equivalent age (TEA). For most of the items, the responses were similar at both ages, while for four items (vertical and arc tracking, ability to discriminate striped black/white targets, and attention at distance), the responses were better at TEA, indicating some maturation in the responses.

A review by Mercuri et al. [18] summarized neurological findings but also visual developments in preterm (both LP and VP) and healthy term-born during the neonatal period assessed using the HNNE and a battery for visual assessment and concluding that these assessments can be easily and reliably used in both very and late preterm infants with in general more immature responses than full-terms.

### 3.2. Studies Reporting Data after the Neonatal Period, in the First Two Years after Birth

Seven papers [1,19,20,21,22,23,24] were included in this group, most of them using the Hammersmith Infant Neurological Examination (HINE) (*n* = 3) or the GMA (*n* = 2). One was a review study [1]. Papers are summarized in Table 2.

One study used the HINE in 448 low-risk LP (50% males) born between 35 and 36 weeks GA, assessed at 6, 9, and 12 months CA [19]. There was a progressive maturation in the items assessing “tone”, “posture” and “reflexes”, but no differences between infants born at 35 and 36 weeks GA. The 10 percentile and the median global HINE score of this cohort were lower than in FT, especially in the sub-sections of tone and reflexes.

In another study [20] from the same group, the HINE was assessed at 3, 6, 9, and 12 months in 188 infants including 71 LP (39 males and 32 females), but also 69 VP (38 males and 31 females), and 48 FT (28 males and 20 females). The results indicated that infants born VP and LP showed significant lower global scores and tone scores than those born FT at each age assessment. Although VP reported a trend of HINE scores generally lower than LP, these differences were not statistically significant. No gender differences were reported on the neurological examinations.

Chatzioanidis et al. [21] also assessed 106 high risk LP (58 males and 48 females) using the HINE at 6 months and 12 months corrected age. At 12 months, they found some differences in the global score and in the posture subscore between infants born at 34 and at 36 weeks, and global score and subscores of posture and reflexes between infants born at 35 and 36 weeks, with more mature findings at older GA. The authors didn’t find any difference between infants born at 34 and 35 weeks. The only factor that significantly influenced the global score was being born small for gestational age (SGA).

A commentary to the paper confirmed that LP had a different development during the first 12 months of life compared with FT [1].

Of the two studies using GMA in LP, one [22] assessed writhing and fidgety in a cohort of 574 LP with the purpose of establishing their predictive value for neurodevelopmental outcome. A significant correlation was found between GMA and neuromotor outcome with a very high sensitivity and specificity in predicting the development of CP both at one month (se 100%, sp 86%) and at three months (se 100% sp 97%).

The other study [23] analyzed GMA in 23 LP and MP (14 males and 9 females) at two points of time, first before term age, around 35 and 37 weeks post-menstrual age, and second at the corrected age of three months by using triaxial accelerometers placed on specific positions on the hands and feet. This study demonstrated substantial changes in most of the parameters between the first and the second measurement, in particular spontaneous motility increase in variability and diversity like as the growth of periodicity of velocity of movements; the periodicity of velocity distribution of arm and leg movements became more regular and variable, indicating that infants acquire a greater repertoire of movement patterns at older ages.

Another study specifically assessed the Forward Parachute Reaction (FPR) [24] during the first year of age with the aim of describing its development in a cohort of 484 (246 males and 238 females) low-risk near-term infants (born at a gestational age between 35.0 and 36.9 weeks) and to correlate with the age of acquisition of independent walking. The authors found that most of infants showed a complete FPR from nine months with a consequent development and acquisition of independent walking comparable to that of FT. There was another group of infants (the 21% of the cohort) who developed an incomplete FPR only with the acquisition of an independent walking at older ages.

### 3.3. Studies Reporting Incidence of CP and Motor Disorder During the Infancy

Eighteen studies [2,4,8,9,25,26,27,28,29,30,31,32,33,34,35,36,37,38] reported the association between late-preterm birth and cerebral palsy (CP), motor delay and coordination disorder; two were review studies [8,27]. Papers are summarized in Table 3.

Some of these studies were based on national registries. Hirvonen et al. [25] used the Finnish national register, which included all live-born infants from 1991 to 2008, with 965,224 FT, 6799 MP, 39,932 LP, 6347 VP. They found that the LP population had a higher risk of developing CP compared to FT with an odds ratio (OR) of 2.35 (95% confidence interval [CI]: 1.99, 2.77) and an incidence of 0.6% vs. 0.1%. The analysis of CP subtypes showed no statistical differences between the groups according to the GA. Predictive factors for developing CP were birth at an earlier period, being SGA, having asphyxia and the evidence of intracranial haemorrhage. In this study, the diagnosis of CP, based on medical history, ultrasound and magnetic resonance imaging data, was usually performed within the first two years of life and almost confirmed by the age of 3 to 4 years.

A meta-analysis study reported a statistically significant association between SGA and CP in moderate and late preterm infants (OR: 2.34; 95% CI: 1.43–3.82) [26].

In agreement with this study, other authors [4,8,27,28] showed a 3-fold increased risk of CP for LP compared with FT, with a hazard ratio of 2.7 to 3.68 and an incidence of 0.3–0.4% in LP compared with 0.1% in FT. However, the exact age of the diagnosis of CP was not always reported.

Thygesen et al. [29] using the Danish Medical Birth Registry analyzing the incidence of CP in a cohort of LP and MP (21,339 males and 18,081 females), with and without respiratory distress syndrome. They found an increased risk of CP in infants with a respiratory distress syndrome.

In another study [33], the incidence of neurosensory impairments including CP, was evaluated through a questionnaire proposed to parents at two years corrected age. Diagnosis of CP was made in four full-term infants and none in the LP and MP. The low prevalence of CP did not allow to evaluate the significance of group differences.

Four studies focused on CP and motor delay and coordination disorder. Odd et al. [30] studied 741 LP and MP (422 males and 319 females) at the age of 7–8 years assessed by ALSPAC coordination test, which consists of three of the eight subtests of the Movement Assessment Battery for Children, to test the three realms of coordination: manual dexterity (placing pegs task), ball skills (throwing bean bag into box), and balance (heel-to-toe walking); Children born moderate to late preterm were more likely to be diagnosed with CP than full-term born (0.9% vs. 0.1%, *p* < 0.001) and reported a higher incidence of coordination problems (3.5% vs. 2.2%, *p* = 0.017). However, when restricting the analysis to those infants at low risk only, LP and MP showed minimal differences to FT, suggesting that the outcome was mainly determined by antenatal and intrapartum factors.

Coordination disorder was also explored in a population of 68 LP (45 males and 23 females) (mean age 7.5 years), [2]. The incidence of was 19.1% according to the Diagnostic and Statistical Manual of Mental Disorders Fifth edition (DSM-5) criteria with an association to other neurodevelopmental disorders like as language delay and Attention Deficit Hyperactivity Disorder.

Jia You et al. [9] analyzed 112 LP (61 males and 51 females) compared to 179 FT (85 males and 94 females) using the motor skills items of Chinese versions of the Gesell Development Diagnosis scale and a neurological examination assessed between 2 and 2.5 years; the two groups were significantly different in terms of motor disorders with LP showing a motor disorder in 9.82% (two cases of CP, nine cases of motor delay) whereas FT in 0.56% only.

Motor skills were also assessed at a mean age of 16.5 months using Bayley scales of infant and toddler development, version III (BSITD III) in 56 LP infants (21 males and 35 females) [31], although the 7.1% of this population had evidence of developmental disability (two cases of CP and four diagnosed as disabled), no statistically significant difference in the overall performance scores were observed in comparison to typically developed control infants.

Conversely, in another study, Morag et al. [32] assessed LP and FT motor competence with Alberta Infant Motor Scale (AIMS) at six months of age and with the Griffiths Mental Development Scales (GMDS) at 12 months of chronological age. Infants born late-preterm reported significantly lower scores in all subscales, but when scores were corrected for prematurity, developmental scores were similar in the two groups.

The following studies [34,35,36,37,38] focused on developmental delay, using the Ages and Stages Questionnaire (ASQ), which explore motor domains (fine and gross motor competence) and other domains (communication, problem-solving and personal-social skills).

The first two studies [34,35] used data from the Longitudinal Preterm Outcome Project (Lollipop). Potijk et al. [34] assessed the effects of low socioeconomic status (SES) and moderate-late prematurity on preschool developmental delay. They included 926 MP and LP infants and 544 FT infants, in which the developmental outcomes were measured using the Dutch version of the 48-month form of the ASQ. Results showed that decreasing gestational age was associated with an increased risk of delay in general development, motor and communication skills. In conclusion, low socioeconomic status and moderate-late prematurity are separately associated with developmental delay with multiplicative effects.

The same questionnaire (ASQ) was used in another study, performed by Hornman et al. [35] with the aim of assessing the stability of developmental problems in MP and LP children compared with VP and FT children, before school entry, at four years of age and one year after school entry, at five years of age. They included 376 VP (191 M, 185 F), 688 MP, and LP (401 M, 287 F) and 403 FT (190 M, 213 F) children. The ASQ-4 total score was abnormal more frequently in all preterm group than in FT group, while the total ASQ-5 score was abnormal more frequently in VP children only and not in MP-LP children. The stability of the ASQ total score of MP and LP children was comparable with FT children; instead, in the VP group, there was a higher rate of emerging problems and persistence of development problems. The whole preterm group showed more emerging motor problems and more resolving communication problems.

Benzies et al. [36] performed a prospective cohort study, including 82 LP (45 M, 37 F) children evaluated with ASQ at 4, 8, and 18 months corrected age. LP group showed lower scores in fine motor domain at four months of age, in gross motor and communication domains at eight months of age. Instead, there were no significant differences with normative sample at 18 months of age.

The same questionnaire was used in the study performed by Ballantyne [37] who analyzed a population of 52 LP infants (30 M, 19 F) and 156 FT infants (95 M, 61 F) at 12 months of age. Results showed that LP infants had an increased risk of developmental delay in the gross motor and communication domains. This difference was reduced when adjustments were made for intensive neonatal care admission.

Finally, in the study performed by Schonhaut [38] who used the same questionnaire, LP infants (89 M e 76 F) showed an increased impairment in fine and gross motor domains.

## 4. Discussion

The present review underlines on one hand the higher risk of neurological impairments in LP than full-term infants, and on the other that LP could follow a specific developmental trend during the first years of age. This applies not only to the high risk LP children but also to the low risk ones with normal imaging and without any other significant risk factors than prematurity. This is particularly true during the first 12 months of life probably due to a brain immaturity and an increased vulnerability to injury, as the last six weeks of gestational age are essential for the cortical grey and white matter development [39]. Not surprisingly, the presence of brain lesions in high risk LP infants is an additional negative prognostic factor on their development both during the first year CA and later in life.

LP infants are considered as a “population at risk” due to a greater risk of neonatal morbidities and mortality than FT [40]. However, most of the reviews of the literature reported on the cognitive and language development only and no-one explored specifically the early neuromotor outcome in this population of infants.

The differences among different levels of prematurity within the LP group, as measured from GA, and with the full-term infants, were mainly detected on the most commonly used structured neurological assessments such HNNE and the GMs [10,18,22], each contributing to highlighting differences in different aspects of neurological function. The use of the HNNE highlighted that low-risk LP newborns showed a more immature findings than FT in tone, spontaneous motility, visual functions and in some reflexes (Moro reflex) items, especially for those born at 34 week GA. This could be observed also during the first year, as, although LP showed a progressive improvement of neurological findings, they continued to report more immature responses than FT in the same neurological item (tone, posture, movements and reflexes), especially in infants born at 34 GA and for those born SGA [19,20,21]. These findings were similar to those obtained in very preterm infants, who also had differences in tone, posture, and reflexes compared to term ones [19,20]; these data suggest that low-risk preterm infants follow a specific neurological development during the first year of age, irrespective to GA, with minimal differences between VP an LP, and that some clinical patterns like as tone, posture, and reflexes should be considered as discriminating patterns between preterm and term born infants in early infancy. Even two recent reviews of the literature, exploring the general neurological, neurovisual and neurobehavioral assessment of LP confirmed immature performances compared to FT infants [14,18].

The findings were also confirmed when assessing specific aspects of neurological assessments in LP infants, such as the FPR [24]. The development of the complete FPR, within 12 months of age, was strictly associated to the acquisition of independent walking in LP similar to that of FT. The presence of an incomplete FPR only was related with a delay in the independent walking, which could be considered the expression of an immature pattern of sensory-motor development.

In the studies assessing GMs, the authors reported the description of the development in low and high-risk LP infants but no specific difference between FT and LP was explored [22,23].

This tool was also studied to identify as prognostic indicators of future outcome. GMs assessed at one and three months confirmed to show a very high power prediction for the development of CP even in a population of LP and mainly when assessed at three months using the fidgety movements, probably due to a progressive improvement and substantial changes in most of the parameters like as variability and velocity of movements at this ages.

Studies exploring outcomes confirmed that LP infants were more likely to present cerebral palsy than infants born at term, with an incidence of 0–3–0.9% compared to 0.1% in FT; however, in a few of them, the diagnosis of CP was based on a single neuroimaging with no mention on clinical assessments and a definite conclusion is not possible for this group of studies due to the controversies about the predictive abilities of conventional neuroimaging [41].

Other motor disorders were explored as coordination problems with an incidence between 10–19.1% in LP.

The high occurrence of motor and neurodevelopmental sequelae is probably related to the neuroanatomical characteristics found in LP and to the high incidence of brain abnormalities, as periventricular hyperechogenicity, IVH, cPVL, that, especially in LP at lower GA, are more similar to VP than to FT infants [42,43] and that increased the risk of motor impairment like as CP [25]. The difference with FT infants was also observed when grey matter volume was assessed, as this was significantly smaller especially in basal ganglia, thalami, and cerebellum. Although an increased risk for neonatal morbidity in LP male gender infants is widely reported in literature, gender differences were not consistently observed on neurological outcome.

One of the strengths of the present review is that it included specific early neuromotor assessments like the HNNE, HINE, and GMs, considered as predictors of early detection of CP even in LP infants especially when integrated with the use of MRI [44]. Our data could be therefore useful for those clinicians involved in the follow-up of LP infants and for families who always require information on the development of his child.

We acknowledge that there are methodological issues that may have affected the analysis of the reviewed papers, such as the presence of few studies reporting the data of both late and moderate preterms in the global analysis; in those studies, it was impossible to extract the data of late-preterm only. However, in the few studies [12,25,30] in which the authors compared the two population of infants, minimal differences were reported.

Other potential limitations are that most of the studies were done more than 10–15 years ago, had a relative low number of infants included, and had a heterogeneity of the methods used for neurological assessments.

## 5. Conclusions

Even with these limitations, the analysis of the studies confirmed that LP infants show a global immature neuromotor development compared to FT especially at early ages. Gestational appropriate reference data should be used in low-risk LP infants as they could be reported as having problems if the criteria used in term born infants were applied. This could also help to better identify possible abnormal signs that should be further investigated for a possible early treatment in LP at risk of neurological impairments.

## Figures and Tables

**Table 1:** Studies reporting neonatal neurological assessment.

Authors	No. of Patients	Ga (Weeks)	M:F	Age of Assessment	Tool	Main Results
Romeo et al. [10]	375	34–36	1.09	Term-age	HNNE	Differences in scoring among the GAs
Romeo et al. [3]	118	34–36	1.18	48–72 h after birth	HNNE	Similar distribution of the scores among the GAs
Chin et al. [11]	79	34–36	1.19	12–72 h after birth and at term-age	HNNE	Differences in ‘tone’ and ‘movement’ items between LP and FT
Spittle et al. [12]	129	34–36	1	2 days–2 weeks after birth	HNNE, NNNS, GMA	Lower scores in most of the items for LP population compared to FT
Spittle et al. [13]	197	32–36	0.95	Term-age and at 2 years CA	HNNE, NNNS, Bayley Scales	Low scores on HNNE and NNNS were associated with cognitive delay
Cheong et al. [14]	NR	34–36	NR	NR	Review: newborn neurobehavioral assessments	Suboptimal scores in LP compared with FT
Eeles et al. [15]	196	32–36	0.9	Term-age	HNNE, NNNS, GMA	Significant relationship between MRI abnormalities and suboptimal scores on the NNNS and the HNNE, and abnormal GMA
Ince et al. [16]	35	34–36	0.59	48 h of birth	Moro reflex	Significant difference for latency interval in the complete Moro response between LP and FT
Romeo et al. [17]	80	34–36	0.9	48–72 h after birth and at term-age	Neurovisual functions	More mature skills in LP at term-age
Mercuri et al. [18]	NR	34–36	NR	NR	Review: HNNE and neurovisual functions	LP had more immature responses than FT

GA, gestational age; HNNE, Hammersmith Neonatal Neurological Examination; NNNS, Neonatal Intensive Care Unit Network Neurobehavioral Scale; GMA, General Movement assessment; CA, corrected age; NR, not reported; LP, late preterm; FT, full term, MP, moderate preterm; VP, very preterm.

**Table 2:** Studies reporting data after the neonatal period, in the first two years after birth.

Authors	No. of Patients	Ga (Weeks)	M:F	Age of Assessment	Tool	Main Results
Romeo et al. [19]	448	35–36	1	6–9–12 months CA	HINE	HINE score were lower in LT than in FT, especially for tone and reflex items
Romeo et al. [20]	71	33–36	1.2	3–6–9–12 months CA	HINE	Lower global and tone scores in LP than FT
Chatzioanidis et al. [21]	134	34–36	1.2	6–12 months CA	HINE	Differences in scores among the GAs
Romeo et al. [1]	NR	34–36	NR	NR	Review	LP had a different development during the first year of life
Brogna et al. [22]	574	34–36	NR	1–3 months CA	GMA	Significant correlation between GMA and neuromotor outcome
Bultmann et al. [23]	23	32–36	1.5	Term-age and at 3 months CA	GMA	Substantial changes in most of the parameters between the first and the second measurement
Romeo et al. [24]	484	35–37	1.03	3–6–9–12 months CA	Forward parachute reaction (FPR)	Similar development between LP and FT

GMA, General Movement assessment; HINE, Hammersmith Infant Neurological Examination; GA, gestational age; CA, corrected age; NR, not reported; LP, late preterm; FT, full term, MP, moderate preterm; VP, very preterm.

**Table 3:** Studies reporting incidence of CP and motor disorder during the infancy.

Authors	No. of Patients	Ga (Weeks)	M:F	Age of Assessment	Tool	Main Results
Hirvonen et al. [25]	39,932	34–36	NR	First 4 years of life	Neuro-imaging and clinical multidisciplinary evaluation	LP had a higher risk of developing CP compared to FT
Zhao et al. [26]	135,650	32–36	NR	NR	Neuro-imaging and clinical multidisciplinary evaluation	Significant association between small for gestational age and CP in LP
Petrini et al. [4]	8341	34–36	1.19	NR	ICD-9	3-fold increased risk of CP in LP infants population
Teune et al. [8]	40,416	34–36	NR	NR	Review	LP had a higher risk of developing CP compared to FT
Favrais et al. [27]	NR	34–36	NR	NR	Review	LP had a higher risk of developing CP compared to FT
Moster et al. [28]	31,169	34–36	1.22	NR	National registry	LP had a higher risk of developing CP compared to FT
Thygesen et al. [29]	39,420	32–36	1.18	First 5 years of age	National Cerebral Palsy Registry	Increased risk of CP in infants with respiratory distress syndrome
Johnson et al. [33]	638	32–36	1.16	2 years CA	Questionnaire proposed to parents	No difference in incidence of CP between LP and FT
Odd et al. [30]	741	32–36	1.3	7–8 years of age	ALSPAC coordination test	Higher incidence of CP and coordination problems in LP
Palumbi et al. [2]	68	34–36	1.95	Mean age: 7.5 years	DSM-5	Coordination disorder in 19.1% of LP
You et al. [9]	112	34–36	1.19	2–2.5 years	Chinese versions of the Gesell Development Diagnosis scale and a neurological examination	Higher incidence of motor disorder in LP than FT
Ramdin et al. [31]	56	34–36	0.6	Mean age: 16.5 months	Bayley scales	No difference between LP and FT
Morag et al. [32]	124	34–36	1.06	6 and 12 months	Alberta Infant Motor Scale and Griffiths Scales	LP had lower scores in all subscales then FT, using CA
Potijk et al. [34]	926	32–35	NR	2 years	ASQ	Decreasing gestational age was associated with an increased risk of delay in general development, motor and communication skills
Hornman et al. [35]	688	32–35	1.4	4 and 5 years	ASQ	MP and LP showed a trend comparable with FT. The whole preterm group had more emerging motor problems.
Benzies et al. [36]	82	34–36	1.2	4, 8 and 18 months CA	ASQ	LP had lower scores in fine motor domain at 4 months CA, in gross motor and communication domains at 8 months CA, but no significant differences with normative sample at 18 months CA.
Ballantyne et al. [37]	52	34–36	1.5	12 months	ASQ	LP had an increased risk of developmental delay in the gross motor and communication domains.
Schonhaut et al. [38]	165	34–36	1.1	8 and 18 months	Chilean validated version of the ASQ	LP showed increased impairment in fine and gross motor domains

GA, gestational age; CA, corrected age; NR, not reported; LP, late preterm; FT, full term, MP, moderate preterm; VP, very preterm; CP, cerebral palsy; ICD, international classification of diseases; ASQ, Ages and stages Questionnaires.

## References

[1] Romeo D.M., Brogna C., Mercuri E. (2018). Neurological assessment of late-preterm infants during the first year of age. Eur. J. Paediatr. Neurol..

[2] Palumbi R., Peschechera A., Margari A., Craig F., Cristella A., Petruzzelli M.G., Margari L. (2018). Neurodevelopmental and emotional-behavioral outcomes in late-preterm infants: An observational descriptive case study. BMC Pediatr..

[3] Romeo D.M., Luciano R., Corsello M., Ricci D., Brogna C., Zuppa A., Romagnoli C., Mercuri E. (2013). Neonatal neurological examination of late preterm babies. Early Hum. Dev..

[4] Petrini J.R., Dias T., McCormick M.C., Massolo M.L., Green N.S., Escobar G.J. (2009). Increased risk of adverse neurological development for late preterm infants. J. Pediatr..

[5] Cheong J.L., Doyle L.W., Burnett A.C., Lee K.J., Walsh J.M., Potter C.R., Treyvaud K., Thompson D.K., Olsen J.E., Anderson P.J. (2017). Association Between Moderate and Late Preterm Birth and Neurodevelopment and Social-Emotional Development at Age 2 Years. JAMA Pediatr..

[6] Woythaler M.A., McCormick M.C., Smith V.C. (2011). Late preterm infants have worse 24-month neurodevelopmental outcomes than term infants. Pediatrics.

[7] Morse S.B., Zheng H., Tang Y., Roth J. (2009). Early school-age outcomes of late preterm infants. Pediatrics.

[8] Teune M.J., Bakhuizen S., Bannerman C.G., Opmeer B.C., van Kaam A.H., van Wassenaer A.G., Morris J.M., Mol B.W.J. (2011). A systematic review of severe morbidity in infants born late preterm. Am. J. Obstet. Gynecol..

[9] You J., Yang H., Hao M., Zheng J. (2019). Late Preterm Infants’ Social Competence, Motor Development, and Cognition. Front. Psychiatry.

[10] Romeo D.M., Ricci D., Brogna C., Cilauro S., Lombardo M.E., Romeo M.G., Mercuri E. (2011). Neurological examination of late-preterm infants at term age. Eur. J. Paediatr. Neurol..

[11] Chin E.Y.J., Baral V.R., Ereno I.L., Allen J.C., Low K., Yeo C.L. (2019). Evaluation of neurological behaviour in late-preterm newborn infants using the Hammersmith Neonatal Neurological Examination. J. Paediatr. Child Health.

[12] Spittle A.J., Walsh J., Olsen J.E., McInnes E., Eeles A.L., Brown N.C., Anderson P.J., Doyle L.W., Cheong J.L.Y. (2016). Neurobehaviour and neurological development in the first month after birth for infants born between 32–42 weeks’ gestation. Early Hum. Dev..

[13] Spittle A.J., Walsh J.M., Potter C., McInnes E., Olsen J.E., Lee K.J., Anderson P.J., Doyle L.W., Cheong J.L.Y. (2017). Neurobehaviour at term-equivalent age and neurodevelopmental outcomes at 2 years in infants born moderate-to-late preterm. Dev. Med. Child Neurol..

[14] Cheong J.L.Y., Thompson D.K., Olsen J.E., Spittle A.J. (2019). Late preterm births: New insights from neonatal neuroimaging and neurobehaviour. Semin. Fetal Neonatal Med..

[15] Eeles A.L., Walsh J.M., Olsen J.E., Cuzzilla R., Thompson D.K., Anderson P.K.J., Doyle L.W., Cheong J.L.Y., Spittle A.J. (2017). Continuum of neurobehaviour and its associations with brain MRI in infants born preterm. BMJ Paediatr. Open.

[16] Ince D.A., Ecevit A., Yıldız M., Tugcu A.U., Ceran B., Tekindal M.A., Turan O., Tarcan A. (2019). Evaluation of Moro reflex with an objective method in late preterm and term infants. Early Hum. Dev..

[17] Romeo D.M., Ricci D., Serrao F., Gallini F., Olivieri G., Cota F., Romagnoli C., Mercuri E. (2012). Visual function assessment in late-preterm newborns. Early Hum. Dev..

[18] Mercuri E., Ricci D., Romeo D.M. (2012). Neurological and visual assessments in very and late low-risk preterm infants. Early Hum. Dev..

[19] Romeo D.M., Cioni M., Guzzetta A., Scoto M., Conversano M., Palermo F., Romeo M.G., Mercuri E. (2007). Application of a scorable neurological examination to near-term infants: Longitudinal data. Neuropediatrics.

[20] Romeo D.M., Brogna C., Sini F., Romeo M.G., Cota F., Ricci D. (2016). Early psychomotor development of low-risk preterm infants: Influence of gestational age and gender. Eur. J. Paediatr. Neurol..

[21] Chatziioannidis I., Kyriakidou M., Exadaktylou S., Antoniou E., Zafeiriou D., Nikolaides N. (2018). Neurological outcome at 6 and 12 months corrected age in hospitalised late preterm infants -a prospective study. Eur. J. Paediatr. Neurol..

[22] Brogna C., Romeo D.M., Cervesi C., Scrofani L., Romeo M.G., Mercuri E., Guzzetta A. (2013). Prognostic value of the qualitative assessments of general movements in late-preterm infants. Early Hum. Dev..

[23] Bultmann C.S., Orlikowsky T., Häusler M., Trepels-Kottek S., Disselhorst-Klug C., Schoberer M. (2019). Spontaneous movements in the first four months of life: An accelerometric study in moderate and late preterm infants. Early Hum. Dev..

[24] Romeo D.M., Cioni M., Scoto M., Palermo F., Pizzardi A., Sorge A., Romeo M.G. (2009). Development of the forward parachute reaction and the age of walking in near term infants: A longitudinal observational study. BMC Pediatr..

[25] Hirvonen M., Ojala R., Korhonen P., Haataja P., Eriksson K., Gissler M., Luukkaala T., Tammela O. (2014). Cerebral palsy among children born moderately and late preterm. Pediatrics.

[26] Zhao M., Dai H., Deng Y., Zhao L. (2016). SGA as a Risk Factor for Cerebral Palsy in Moderate to Late Preterm Infants: A System Review and Meta-analysis. Sci. Rep..

[27] Favrais G., Saliba E. (2019). Neurodevelopmental outcome of late-preterm infants: Literature review. Arch. Pediatr..

[28] Moster D., Lie R.T., Markestad T. (2008). Long-term medical and social consequences of preterm birth. N. Engl. J. Med..

[29] Thygesen S.K., Olsen M., Østergaard J.R., Sørensen H.T. (2016). Respiratory distress syndrome in moderately late and late preterm infants and risk of cerebral palsy: A population-based cohort study. BMJ Open.

[30] Odd D.E., Lingam R., Emond A., Whitelaw A. (2013). Movement outcomes of infants born moderate and late preterm. Acta Paediatr..

[31] Ramdin T., Ballot D., Rakotsoane D., Madzudzo L., Brown N., Chirwa T., Cooper P., Davies V. (2018). Neurodevelopmental outcome of late preterm infants in Johannesburg. South Africa. BMC Pediatr..

[32] Morag I., Bart O., Raz R., Shayevitz S., Simchen M.J., Strauss T., Zangen S., Kuint J., Gabis L. (2013). Developmental characteristics of late preterm infants at six and twelve months: A prospective study. Infant Behav. Dev..

[33] Johnson S., Evans T.A., Draper E.S., Field D.J., Manktelow B.N., Marlow N., Matthews R., Petrou S., Seaton S.E., Smith L.K. (2015). Neurodevelopmental outcomes following late and moderate prematurity: A population-based cohort study. Arch. Dis. Child Fetal Neonatal Ed..

[34] Potijk M.R., Kerstjens J.M., Bos A.F., Reijneveld S.A., de Winter A.F. (2013). Developmental Delay in Moderately Preterm-Born Children With Low Socioeconomic Status: Risks Multiply. J. Pediatr..

[35] Hornman J., de Winter A.F., Kerstjens J.M., Bos A.F., Reijneveld S.A. (2017). Stability of Developmental Problems After School Entry of Moderately-Late Preterm and Early Preterm-Born Children. J. Pediatr..

[36] Benzies K.M., Magill-Evans J., Ballantyne M., Kurilova J. (2017). Longitudinal Patterns of Early Development in Canadian Late Preterm Infants: A Prospective Cohort Study. J. Child Health Care.

[37] Ballantyne M., Benzies K.M., McDonald S., Magill-Evans J., Tough S. (2016). Risk of Developmental Delay: Comparison of Late Preterm and Full Term Canadian Infants at Age 12 Months. Early Hum. Dev..

[38] Schonhaut L., Armijo I., Pérez M. (2015). Gestational Age and Developmental Risk in Moderately and Late Preterm and Early Term Infants. Pediatrics.

[39] Adams-Chapman I. (2006). Neurodevelopmental outcome of the late preterm infant. Clin. Perinatol..

[40] Engle W.A., Tomashek K.M., Wallman C. (2007). Committee on Fetus and Newborn, American Academy of Pediatrics. “Late-preterm” infants: A population at risk. Pediatrics.

[41] De Vries L.S., van Haastert I.C., Benders M.J., Groenendaal F. (2011). Myth: Cerebral palsy cannot be predicted by neonatal brain imaging. Semin. Fetal Neonatal Med..

[42] Fumagalli M., Ramenghi L.A., De Carli A., Bassi L., Farè P., Dessimone F., Pisoni S., Sirgiovanni I., Groppo M., Ometto A. (2015). Cranial ultrasound findings in late preterm infants and correlation with perinatal risk factors. Ital. J. Pediatr..

[43] Walsh J.M., Doyle L.W., Anderson P.J., Lee K.L., Cheong J.L.Y. (2014). Moderate and late preterm birth: Effect on brain size and maturation at term-equivalent age. Radiology.

[44] Morgan C., Romeo D.M., Chorna O., Novak I., Galea C., Del Secco S., Guzzetta A. (2019). The Pooled Diagnostic Accuracy of Neuroimaging, General Movements, and Neurological Examination for Diagnosing Cerebral Palsy Early in High-Risk Infants: A Case Control Study. J. Clin. Med..

